# An fMRI investigation of expectation violation in magic tricks

**DOI:** 10.3389/fpsyg.2015.00084

**Published:** 2015-02-04

**Authors:** Amory H. Danek, Michael Öllinger, Thomas Fraps, Benedikt Grothe, Virginia L. Flanagin

**Affiliations:** ^1^Division of Neurobiology, Department Biology II, Ludwig-Maximilians-Universität MünchenMunich, Germany; ^2^Parmenides FoundationMunich, Germany; ^3^Department of Psychology, Ludwig-Maximilians-Universität MünchenMunich, Germany; ^4^Trick 17 Magic ConceptsMunich, Germany; ^5^German Center for Vertigo and Balance Disorders, University Hospital Munich-GroßhadernMunich, Germany

**Keywords:** expectation violation, magic, fMRI, caudate nucleus, perceptual prediction error, movement observation, action

## Abstract

Magic tricks violate the expected causal relationships that form an implicit belief system about what is possible in the world around us. Observing a magic effect seemingly invalidates our implicit assumptions about what action causes which outcome. We aimed at identifying the neural correlates of such expectation violations by contrasting 24 video clips of magic tricks with 24 control clips in which the expected action-outcome relationship is upheld. Using fMRI, we measured the brain activity of 25 normal volunteers while they watched the clips in the scanner. Additionally, we measured the professional magician who had performed the magic tricks under the assumption that, in contrast to naïve observers, the magician himself would not perceive his own magic tricks as an expectation violation. As the main effect of magic – control clips in the normal sample, we found higher activity for magic in the head of the caudate nucleus (CN) bilaterally, the left inferior frontal gyrus and the left anterior insula. As expected, the magician’s brain activity substantially differed from these results, with mainly parietal areas (supramarginal gyrus bilaterally) activated, supporting our hypothesis that he did not experience any expectation violation. These findings are in accordance with previous research that has implicated the head of the CN in processing changes in the contingency between action and outcome, even in the absence of reward or feedback.

## INTRODUCTION

A deep need of humans is to predict future events. This ability, technically speaking causal reasoning, helps us to navigate in a complex world. Although it is questioned whether our conscious will actually controls our actions (Wegner, 2002), it is clear that the perception of causality exists. Evidence from developmental psychology tells us that infants can discriminate causal from non-causal events (Michotte, 1963). In so-called violation-of-expectation tasks, even young infants try to predict the outcome of observed events as evidenced by their looking longer at trials which violate their expectations (e.g., Wang et al., 2004). Over time, humans acquire a broad knowledge base that is constantly enlarged, modified, and updated. Relying on prior knowledge is helpful for learning, for problem solving, for decision making and for more effective action selection (e.g., Ericsson et al., 1993; Ericsson and Lehmann, 1996; Bilalić et al., 2012). To a large extent, this knowledge base consists of the knowledge of causal relations between action and outcome. Long-established causal relations like this one are typically no longer questioned, and not even explicitly represented. This makes the case of magic so interesting: predictions about the outcome of observed actions and violations of these predictions are key ingredients in magic. Magic tricks provide counterfactual evidence to our prior knowledge about objects, how they can be handled and about the set of possible outcomes. Let us consider the following magic trick: sitting at a table, the magician takes an egg from an egg box. He throws it on the floor – and it jumps back into his hands, undamaged. To prove that it is a real egg, he then breaks it and empties the content into a glass. This is astonishing. We have learnt, probably from our own experience, that if we throw an egg to the floor, it will break and not jump. The observed event strongly violates the expected relationship between action (throwing egg to the floor) and outcome (broken egg).

Before we discuss the possible neural basis of the violation-of-expectation that is present in magic tricks, a short clarification of terms is needed. The term “expectation violation” is used in different contexts from developmental psychology (e.g., Wang et al., 2004) and neuroeconomics (e.g., Chang and Sanfey, 2009) to motor control (e.g., Grush, 2004), and thus refers to very different types of expectations. For the purposes of this paper, we define “expectation violation” as the violation of the expected action outcome in a magic trick. This means, the observer watches an entire action sequence and expects a certain outcome – but another outcome is presented.

The brain areas recruited for expectation violation reflect the nature of the task at hand (Bubic et al., 2009). Thus, an anatomical hypothesis can be derived from the very first (and only) study that investigated hemodynamic activity during magic tricks. Contrasting magic tricks with situations in which the expected relationship between action sequence and effect was upheld, Parris et al. (2009) reported activity in the dorsolateral prefrontal cortex (DLPFC) and the anterior cingulate cortex (ACC). The ACC is a key area known to mediate cognitive conflict (e.g., Kerns et al., 2004). This fits with results from another fMRI study that found ACC activated when inconsistent information was presented (Fugelsang and Dunbar, 2005). This is supported by several electrophysiological studies (e.g., Holroyd, 2004), for example Huster et al. (2010) reported the cingulate cortex to be the neural generator of the N200, the event-related potential reliably triggered by Go/Nogo tasks (e.g., Johnstone et al., 2007). However, we believe that Parris et al.’s (2009) study cannot fully answer the question of what brain regions support magic trick expectation violations because their analysis was restricted to only one time point (the discrete time point of the moment of surprise). We argue that although the moment of expectation violation is traceable to a specific time point, expectations related to the magic trick are built up over the entire clip. In order to have expected motor outcomes violated, the entire sequence of preceding events is also taken into account. Otherwise magicians would only have to present one specific movement as a “trick” and not the sequence of movements leading up to the single event that violates the already built-up expectancy. For example, in the magic trick described above, the action of breaking the egg and emptying its content into a glass would not violate any expectations, if the egg had not previously been tossed to the floor and jumped up again. It is possible that different but overlapping cognitive processes are active throughout the entire magic trick and at the specific moment of surprise. For this reason, we decided to also look at the complete time window of each clip, besides analyzing the specific time point of surprise.

Another possible candidate region that could subserve the function of signaling expectation violation is the caudate nucleus (CN). Tricomi et al. (2004) conducted a series of fMRI experiments to disentangle reward-related caudate activity and found that the CN was only active in tasks with a perceived contingency between action and outcome. If the outcome was thought to be unrelated to the previous action, CN was not active. A comprehensive review (including anatomical, behavioral, and imaging studies on healthy controls and patients as well as on animals; Grahn et al., 2008) focusing on the head of the CN sketches its cognitive functions as follows: in contrast to the putamen that is thought to be responsible for more rigid habit learning, the CN is responsible for flexible action-outcome learning, in particular when task contingencies change. It subserves a goal-directed response system that monitors the outcome of an action and responds to changes in the contingency between action and outcome. As discussed, magic tricks overturn the learnt contingencies between initial action and expected effect. We expect that this mismatch will activate the CN.

The aim of the present study is to replicate parts of a previous study using a similar paradigm (Parris et al., 2009) with a larger set of magic tricks (24 instead of 13) and a stronger magnet (3 Tesla instead of 1.5). In contrast to the previous study (Parris et al., 2009), we were additionally interested in ongoing activity throughout the entire magic trick, which should correlate with the build-up of an expectation about the contingency between action and outcome. To further investigate the expectation violation in magic tricks, we measured the professional magician (Thomas Fraps) that had performed the magic tricks, as a single case baseline. In order to be able to flawlessly present magic effects, magicians invest in many years of training. The “choreography,” i.e., the secret as well as the official action sequence of each specific trick must be learnt through many repetitions. Depending on the difficulty of the trick and the experience of the magician, a conservative estimate by Thomas Fraps is that 150–200 repetitions are required. The individual gestures are also practiced separately. We therefore assumed that, in contrast to the naïve observer, the magician himself should not show any expectation violation due to his familiarity with the entire action sequence of each trick. We hypothesize that the magician’s brain activity will differ from that of the experimental group. Contrasting events that violate action-outcome expectations with control events without expectation violation, we hypothesize to find higher activity in the CN, the DLPFC, and the ACC.

## MATERIALS AND METHODS

### PARTICIPANTS

Twenty five healthy right-handed adults (mean age: 26 years, range 21–35 years; 10 male) participated in this experiment. In addition, the right-handed magician that created the magic tricks (male, age 46) also participated in the study. Before beginning the experiment, participants were given a detailed informed consent form describing the study, as well as discomforts and potential risks of functional MRI. After agreeing to participate in the study, participants were additionally orally instructed about the details of their task. Participants were monetarily compensated for their time. Participants had no history of neurological disease, and were not taking medication at the time. All participants understood the instructions without difficulty. Participants had no knowledge of the solutions to the magic tricks at the time of the experiment and had no expertise as magicians. The study was performed in accordance with the Declaration of Helsinki and approved by the ethics committee of the medical faculty of the Ludwig-Maximilians-Universität Munich. None of the participants were excluded from the analysis.

### TESTING MATERIAL AND TASK

#### Magic tricks

**FIGURE 1:**
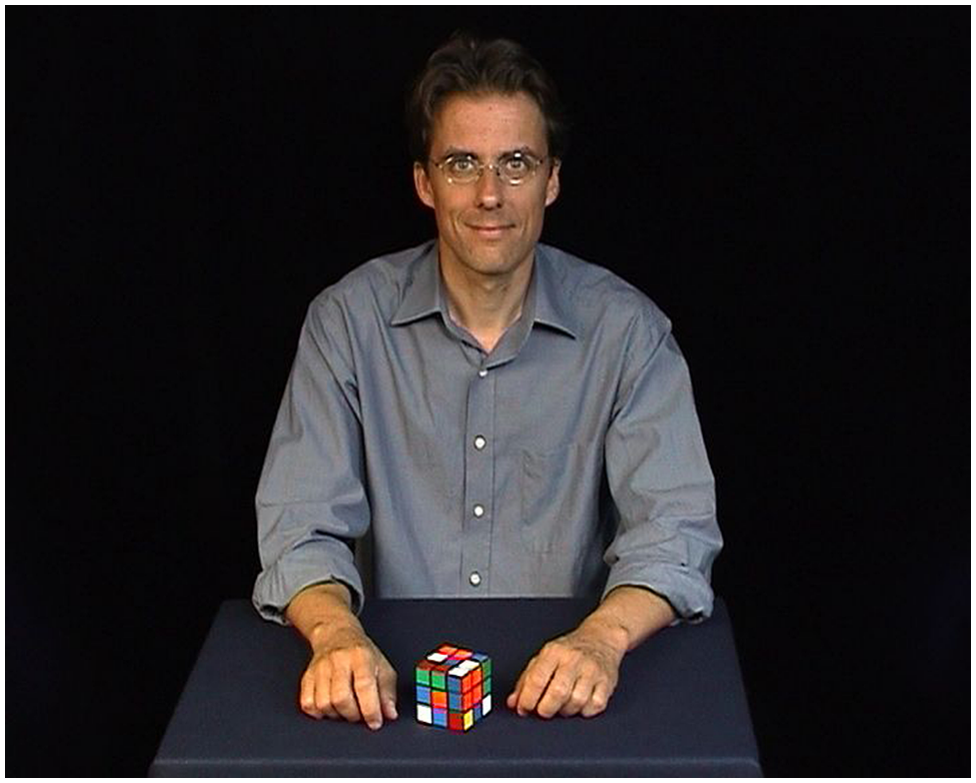
**Standardized setting shortly before the magic trick (here: Rubik’s Cube) is performed**.

We used 24 short video clips of magic tricks, two more clips were shown in the practice trials. They had been performed by a professional magician (Thomas Fraps) and recorded in a standardized theater setting. The magician whose appearance (e.g., shirt) was kept identical during the recording sessions was shown on stage, either seated behind or standing behind a table, see **Figure 1**. The background was a black curtain. The set of tricks included different magic effects (e.g., appearance, levitation, restoration, vanish) and methods (e.g., sleight of hand, gimmicks, optical illusions) and are described in detail in the Supplementary Material. See http://www.youtube.com/watch?v=3B6ZxNROuNw for a sample trick clip. We used short tricks, with only one effect and one key method. Clip duration ranged from 6.3 to 42.5 s. This set of tricks had previously been tested to ensure that all tricks were understandable, i.e., that participants perceived the intended magic effect. This is an important prerequisite for actually experiencing expectation violations. Further, the tricks consisted only of visual effects that could be performed in absolute silence, with no other interactive elements necessary (e.g., assistant, interaction with the audience). Thus, the fMRI signal was only measured during visual, not auditory processing. Further details about the development of these stimuli can be found in a previous paper (Danek et al., 2014).

#### Control clips

For each magic trick, we provided a corresponding control clip (see full list in the Supplementary Material). We made sure that the same general action sequence was shown, but with no magic effect and thus without expectation violation. For example, in the vanishing coin trick (see list), the magician presents three coins in his hand. He closes the hand, shakes it and opens it to reveal that only two coins are left. In the control clip, the magician presents three coins in his hand. He closes the hand, shakes it and opens it to reveal all three coins. Thus, in the control clip, the expectation that all three coins should be still there is not violated.

#### Piloting the testing material

A pilot study was conducted to ensure that the observed events in the magic clips triggered a feeling of surprise and expectation violation. Fifteen independent observers (that did not take part in the subsequent fMRI study, mean age: 24 years, range 20–27 years; 5 male) watched all clips (the 24 trick clips as well as the 24 control clips, in randomized order) and rated them on a scale from 1 (not at all) to 4 (very much) for how surprising the clip was, how much it involved illusion, how much it violated the law of cause and effect and whether the magician’s actions led to an unexpected outcome. On average, the magic clips were rated as follows: surprise 2.94 (SD = 0.3), illusion 3.15 (SD = 0.3), violation of law of cause and effect 3.16 (SD = 0.3), and unexpected outcome 2.86 (SD = 0.3). In contrast, the control clips were rated much lower: surprise 1.19 (SD = 0.2), illusion 1.03 (SD = 0.1), violation of law of cause and effect 1.03 (SD = 0.1), and unexpected outcome 1.17 (SD = 0.1). These differences between magic and control clips with regard to the ratings were all statistically significant (*t*-tests for repeated measures, all *p* < 0.01). Another sample of 15 participants (one of them had to be excluded as an outlier) was presented with both the magic and the control clips (see below) in randomized order and indicated after each clip whether they had seen a magic trick or not. Collapsed across all clips from the same condition (magic or control), 89.7% of all participants identified the magic clips correctly as magic clips and 98.3% of them correctly identified the control clips as such. Thus, compared to the control clips, participants found the magic tricks more surprising, involving more illusion and unexpected outcomes, more strongly violating the law of cause and effect, and they could distinguish them from the control clips.

#### Color task

We also introduced a cognitive task that had nothing to do with magic tricks, in order to allow activity to return to baseline between blocks, but keep attentional demands at a constant level. A color decision task was presented at the end of each block. Different colored squares (red, orange, yellow, green, blue, and violet) appeared on the screen and participants indicated whether the square was a primary color (red, yellow, blue) or not (primary color = left, other color = right). Directly after their response, the next square appeared. Feedback was provided during training, but not during the experiment.

### PROCEDURE

Stimuli were presented in 24 randomized blocks. Each block consisted of one magic trick and the corresponding control clip, in randomized order. In other words, if the control clip were presented first, then the specific magic trick corresponding to that control clip would follow. This was done to reduce the time between consecutive presentations of the same condition, and to minimize the likelihood that subjects would associate films between blocks. After watching the first clip, participants were already aware of the nature of the second clip, so the order of the clips was taken into account during analysis (see Data Analysis). With this design, the expectation violation related to the magic trick is separable from the expectation of the type of clip presented, since the nature of the magic trick (e.g., vanishing, transposition, physical impossibility etc. – see Supplementary Material) is unknown regardless of whether participants know that a magic trick will be shown.

**FIGURE 2:**
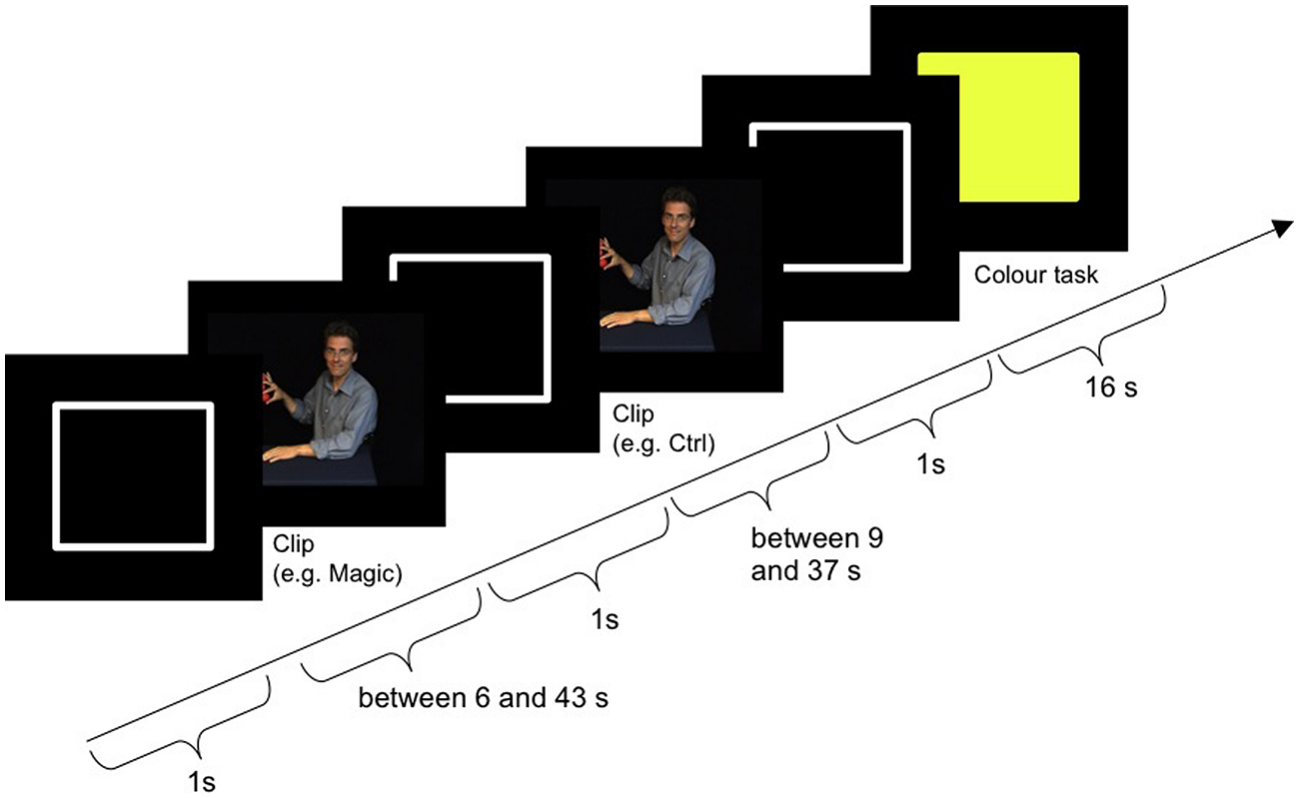
**Procedure of one block including color task**.

**Figure 2** shows the procedure of one block plus subsequent color task. The block started with the outline of a white rectangle (the same size and shape as the video clips) on a black background, which was presented for 1000 ms (±300 ms). Then the magic and the control clip followed in randomized order. The outline was also presented after each clip. Afterward the color task was presented for 16 s between blocks. Subject responses were only required during the color task. For the magic and the control clip, participants were instructed to passively watch the videos. Two practice blocks with feedback were performed outside the scanner. The entire experimental session lasted about 90 min, with 60 min spent in the scanner.

### MATERIAL

Visual stimuli were projected with a LCD projector (Christie LX40, Christie Digital Systems, USA) with a True XGA 1024 × 768 system onto a back-projection screen placed behind participants in the MR-scanner. Participants viewed the projection through a mirror placed 14 cm above them at 45°. The distance from the mirror to the screen was 26 cm for a horizontal visual field of view of 25°. The experiment was run in Matlab 7.5.0 (R2007b, The Mathworks, Inc.) with Cogent Graphics developed by John Romaya at the LON at the Wellcome Department of Imaging Neuroscience. The experiment was controlled from a 64 bit Windows 7 personal computer (Dell Precision M4500) with an NVIDIA Quadro FX 1800M Graphics card.

### fMRI DATA ACQUISITION

Images were acquired with a 3T MRI Scanner (Signa HDx, GE Healthcare, Milwaukee, WI, USA) using a standard 8-channel head coil. Thirty-seven contiguous transverse slices (slice thickness 3.5 mm, no gap) were acquired using a gradient echo echo-planar-imaging (EPI) sequence (TR 2.0 s, TE 40 ms, flip angle 80°. Matrix 64 × 64 voxel, FOV 200 mm). 736 volumes were acquired. After functional imaging, a 3D T1-weighted high-resolution structural image of the entire brain (0.8 × 0.8 × 0.8 isotropic voxel size) was acquired using a fast spoiled gradient recalled sequence.

### DATA ANALYSIS

Functional imaging data were analyzed using Statistical Parametric Mapping (SPM8, Wellcome Department of Imaging Neuroscience, University College London) on Matlab 8.2.0.701 (R2013b). To improve coregistration performance, all images were first manually reoriented so that the origin was set to the anterior commissure. Then the functional volumes were slice time corrected, realigned to the first volume of the first run and then to the mean across all runs. They were then coregistered to the anatomical image from that subject. The anatomical image was segmented into tissue probability maps based on standard stereotaxic space [Montreal Neurological Institute (MNI)], and the transformation parameters used to normalize the functional volumes. Noise was then reduced by smoothing the functional data using a 8-mm full-width at half-maximum Gaussian kernel.

To compare with the previous study (Parris et al., 2009), we determined the discrete time point of the moment of violation of expectancy in the magic trick. These time points were extracted in pilot studies for each trick separately by asking a sample of 15 participants to watch the clips and to quickly press a button in the moment of expectation violation (i.e., the moment where “the magic happens”). Their button press was acknowledged by a short beep. Their reaction times were averaged and used as the time points for the events for the magic clips. For the control clips we took the time points that corresponded to the same relative time than in the magic clip by using the following equation: (surprise moment time divided by entire length of magic clip) multiplied by the length of the control clip. This means that if the expectation violation moment was at 80% of the length of the magic clip, then the event for the control clip was also set to 80% of the control clip.

Functional data were analyzed in each single subject using two univariate multiple regression models. Both models included separate predictors for magic and control clips, separated by the order of appearance within a block (first or last). In the first model, the events were time-locked to the moment of expectation violation and the duration of the event was set to 0 as in the Parris et al. (2009) study. In the second model, we used regressors that were time-locked to the start of the video presentation, with a variable duration depending on the length of the video clip. Each single-subject model therefore included four events of interest corresponding to a 2 × 2 factorial design with factors film type (magic/control) and order (first/last). These events were convolved with the canonical hemodynamic response function (HRF). The six motion correction parameters from the realignment step were modeled separately as events of no interest. The data were high-pass filtered (cutoff frequency = 0.0078 Hz) to minimize slow scanner related drifts and global changes were removed by proportional scaling. For each subject, we computed four contrasts that averaged the parameter estimates across the two fMRI-runs, as a function of condition.

The contrast estimates for each subject and condition were then entered into two whole-brain group-level within-subject 2 × 2 ANOVAs, with the same factors and levels as above, plus participant effects. One ANOVA analyzed the time point of the expectation violation, the other ANOVA modeled the entire clip. All normal subjects were used in both models (*N* = 25). This allowed us to test for main effects of order and film type as well as any interactions. Corrections for non-sphericity accounted for non-independent error terms for the repeated measures as well as differences in error variance. We then tested for differences between the magic tricks and the control clips, both as main effects and as interactions.

We compared the results of the normal healthy group to the single subject results from the magician by calculating the percent of overlapping supra-threshold voxels for the contrast magic-control. In addition, we created a group-level model to test for differences between the magician and the normal participants for the main effect of magic tricks vs. control clips, although the informative value of this analysis is limited due to the group size of one for the magician. Nonetheless, we tested for similarities between the two groups using a conjunction analysis with the conjunction null (Nichols et al., 2005). For comparison with the previous study and to enable meta-analyses, both the images and the tables are presented at a threshold of *p* < 0.001 uncorrected for multiple comparisons and a voxel extent threshold of 30 voxels. However, we consider only voxels that survive a voxelwise statistical threshold of *p* = 0.05 family wise error (FWE) corrected for multiple comparisons across the entire brain volume for further discussion. The *p* < 0.05 FWE corrected *p*-values are presented in the tables. Anatomical regions were identified by manual inspection using the Juelich Histological Atlas and the Harvard Oxford Structural Atlas (in FSLView 3.1.8).

## RESULTS

The results are organized as follows: first, the main effect of expectation violation at the time point of the violation is presented in our experimental sample (*N* = 25) and compared to the findings of a previous study (Parris et al., 2009). Second, the main effect of expectation that exists throughout the entire trick is presented. Third, the individual activity of the magician who performed the tricks will be presented, using the same contrast. Fourth, the findings from the magician will be contrasted with those from the naïve lay sample.

### EXPECTATION VIOLATION (MAGIC – CONTROL): MOMENT OF VIOLATION

To examine the effect of expectation violation, independent of when the film was presented, we examined the main effect of magic tricks vs. control clips, at the moment of magic, determined by independent ratings of each clip (see Materials and Methods). We did not find any supra-threshold voxels for the interactions between film type and order, so we continued to look only at the main effect of film type (magic vs. control). The main difference between the magic tricks and the control clips is the lack of expectation violation in the latter. The same objects are used in a very similar action sequence, but without any unexpected outcome. For example, the magician closes his fist around a silver coin, and when he opens the fist again, the coin is still there, as expected. The standard action-outcome sequence is thus preserved in the control clips.

**FIGURE 3:**
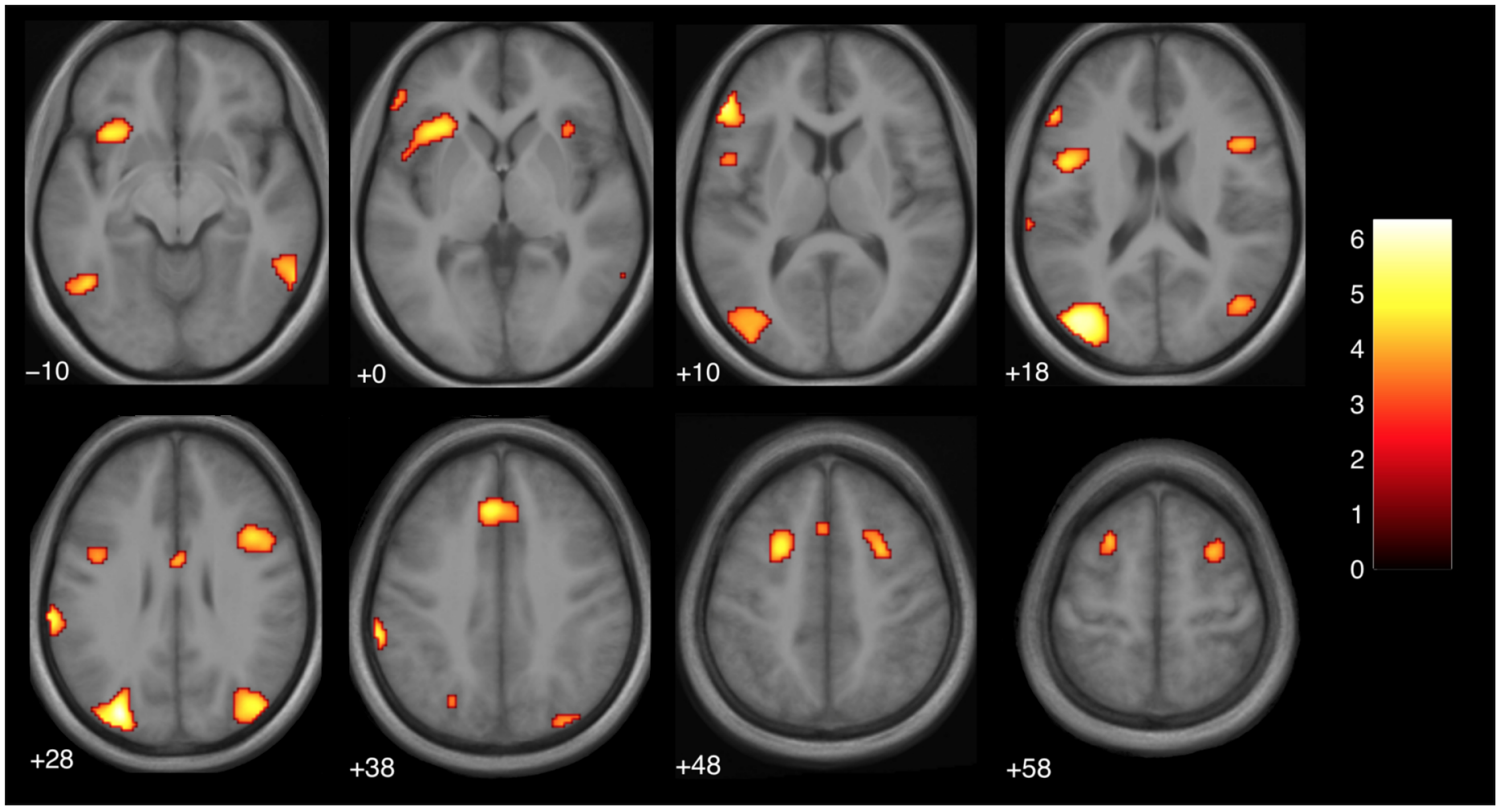
**Brain activity at the moment of expectation violation for magic tricks compared to control clips, independent of presentation order (main effect, *p* < 0.001 uncorrected, voxel cluster threshold 30).** The discrete time point of magic was determined by an independent group of subjects (see Materials and Methods). The color bar depicts the *t*-values of the supra-threshold voxels. Activations are overlaid on the normalized average structural image from all subjects tested, values represent *z*-values in Montreal Neurological Institute (MNI)-coordinates.

In this analysis, we saw a left dominant activity that partially overlapped with those seen in the previous study (Parris et al., 2009). However, unlike Parris et al. (2009), we did not use a region of interest analysis and the regions survive after a more stringent statistical threshold. The regions are reported in **Table 1** and **Figure 3**. The clusters in the inferior frontal gyrus are very similar to those found in an action-observation study (Kilner et al., 2009), which suggests that the action-outcome processing is taking place. The activity in the occipital lobe is known to process visual motion (Greenlee, 1999), which would be involved in understanding the violation of the action-outcome in magic.

**Table 1:** Activation clusters for comparison magic – control for the discrete time point of the moment of magic (i.e., expectation violation).

Anatomical area	*X*	*Y*	*Z*	*k*	*t-*value	*P*_FWE-corr_
Left superior lateral occipital cortex	-30	-80	28	393	6.33	0.000
Left inferior frontal gyrus, pars triangularis	-52	34	10	40	5.67	0.003
Left anterior supramarginal gyrus	-66	-32	32	28	5.47	0.007
Left posterior cingulate gyrus	-4	28	40	26	5.45	0.007
Left anterior insula	-32	20	-4	33	5.18	0.018
Left superior frontal gyrus	-24	10	52	17	5.17	0.019
Right superior lateral occipital gyrus	44	-78	32	12	5.02	0.031
Right middle frontal gyrus	28	8	52	226	4.88	0.049
Right inferior temporal gyrus, temporo-occipital division	62	-56	-8	221	4.79	0.064
Left inferior temporal gyrus, temporo-occipital division	-46	-60	-12	148	4.69	0.088
Anterior cingulate gyrus	0	0	26	34	4.23	0.303
Left amygdala	-22	-8	-20	50	3.78	0.721
Left anterior insula	34	20	-2	53	3.71	0.775

*A voxel cluster threshold 30, *p* < 0.001, uncorrected for multiple comparisons was used, but the *p*-values for a voxel-wise FWE-corrected threshold are shown. Montreal Neurological Institute (MNI) coordinates are used.*

#### Comparison to previous literature

The regions that were more active during a violation in expectation are similar to those found in a previous study with a similar design (Parris et al., 2009). In particular, the DLPFC (superior frontal gyrus), and parts of the cingulate gyrus were active bilaterally (see **Table 1**; **Figure 3**). In the previous study, similar regions were active but in a left-dominant manner. For comparison, results from Parris et al. (2009) are listed in **Table 2**.

**Table 2:** Significant clusters found in Parris et al. (2009) for comparison magic – control.

Anatomical area from Parris et al. (2009)	*X*	*Y*	*Z*
Left superior frontal gyrus	-24	10	58
Left middle frontal gyrus	-22	36	44
Left middle frontal gyrus	-42	23	26
Left anterior cingulate	-4	38	19

*Corresponding areas are marked red. We used the program “tal2mni.m” from http://imaging.mrc-cbu.cam.ac.uk/imaging/MniTalairach to convert their Talairach coordinates to the MNI values reported here.*

### MAGIC – CONTROL: ENTIRE CLIP DURATION

**FIGURE 4:**
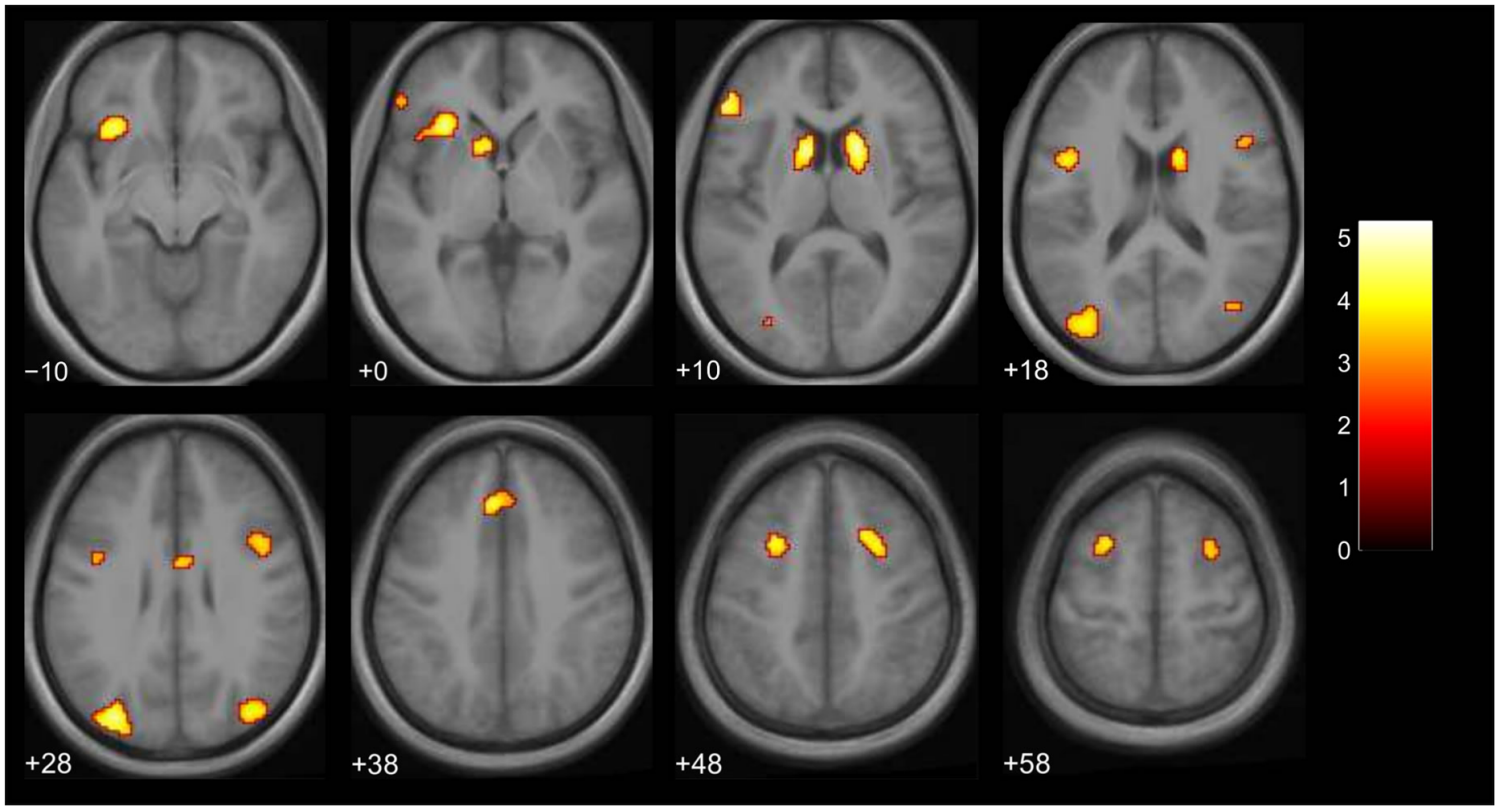
**Brain activity for entire clip duration for the contrast magic – control (main effect, *p* < 0.001 uncorrected, voxel cluster threshold 30).** The color bar depicts the *t*-values of the supra-threshold voxels. Activations are overlaid on the normalized average structural image from all subjects tested, values represent *z*-values in MNI-coordinates.

We then examined the main effect of magic tricks vs. control clips, for the entire duration of the magic clip. By examining the entire clip, regions involved in the expectancy throughout the entire action sequence should be revealed. We found higher activity in four distinct clusters for magic tricks compared to control clips. These were the head of the CN bilaterally, the left inferior frontal gyrus and the left anterior insula (see **Table 3**; **Figure 4**). Additional frontal and occipital regions overlapping with those found at the time point of the violation of expectation were also significantly active at a more liberal threshold.

**Table 3:** Clusters for comparison magic – control throughout the entire clip presentation (voxel cluster threshold 30, *p* < 0.001, uncorrected).

Anatomical area	*X*	*Y*	*Z*	*k*	*t-*value	*P*_FWE-corr_
Right caudate nucleus (CN; head)	14	8	14	21	5.26	0.011
Left CN (head)	-10	12	6	18	5.24	0.011
Left inferior frontal gyrus	-50	32	6	10	5.09	0.019
Left anterior insula	-32	22	-6	12	4.93	0.031
Left lateral occipital cortex, superior division	-32	-80	26	452	4.87	0.038
Right superior frontal gyrus	28	8	54	244	4.66	0.074
Left superior frontal gyrus	-26	10	54	201	4.52	0.111
Left paracingulate gyrus	-6	30	38	138	4.43	0.144
Right lateral occipital cortex, superior division	38	-78	28	170	4.13	0.307
Right anterior cingulate gyrus	4	0	26	58	4.10	0.330
Left inferior frontal gyrus	-44	4	18	135	4.02	0.395
Right inferior frontal gyrus	44	12	24	183	3.91	0.492

*MNI coordinates are used. The *p*-values for a voxel-wise FWE-corrected threshold are shown.*

### LACK OF EXPECTATION VIOLATION: ACTIVITY IN A MAGICIAN

**FIGURE 5:**
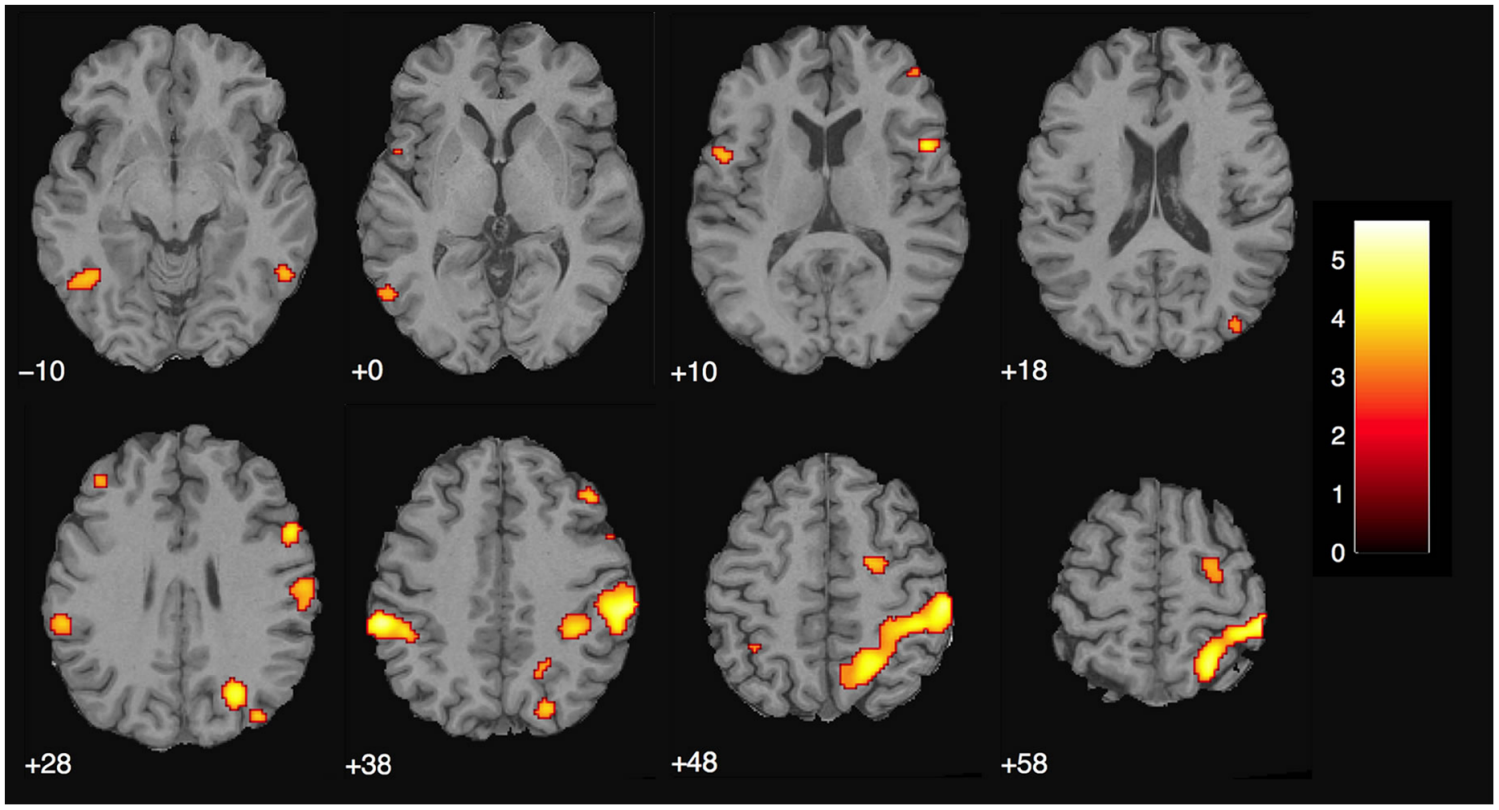
**Magician Thomas Fraps: significant activity for magic vs. control condition showing sensory-motor and parietal activity in the magician**. The color bar depicts the *t*-values of the supra-threshold voxels. Activations are overlaid on the normalized structural image from the magician tested, values represent *z*-values in MNI-coordinates.

We assumed that, in contrast to naïve observers, the magician would not perceive the magic effect as an expectation violation since he had performed the magic himself and knew the entire action sequence of each trick and each control clip, (see Introduction). As expected, the activity in the magician’s brain substantially differed from the activity of our experimental sample. Calculating the same magic vs. control contrast as before, we found significant activity in the parietal lobe, namely in the supramarginal gyrus (which is part of the inferior parietal lobule) bilaterally, in the right superior parietal lobule as well as in the right postcentral gyrus, see **Table 4**; and **Figure 5**.

**Table 4:** Activity in the magician (Thomas Fraps).

Anatomical area	*X*	*Y*	*Z*	*k*	*t-*value	*P*_FWE-corr_
Right supramarginal gyrus	60	-26	44	2709	5.65	0.001
Right superior parietal lobule*	26	-56	56		5.21	0.005
Right postcentral gyrus*	52	-34	58		4.95	0.015
Left supramarginal gyrus	-58	-34	34	394	5.58	0.001
Right precentral gyrus	54	12	32	144	4.73	0.037
Right inferior frontal gyrus, pars opercularis	50	10	12	68	4.48	0.097
Left precentral gyrus	-52	6	6	84	4.27	0.201
Right premotor cortex	24	-4	50	177	4.11	0.332
Right middle frontal gyrus	44	30	42	58	4.05	0.395
Right premotor cortex	14	2	68	98	3.97	0.487
Right superior lateral occipital cortex	40	-80	26	78	3.96	0.500
Left inferior temporal gyrus, temporooccipital division	-44	-58	-10	193	3.74	0.750
Left frontal pole	-42	42	24	59	3.74	0.757
Left inferior temporal gyrus, temporooccipital division	56	-56	-12	51	3.73	0.767
Right frontal pole	42	46	8	55	3.63	0.860
Superior parietal lobe	-34	-54	52	33	3.41	0.976

*Shown are all clusters for comparison magic – control for the entire clip duration (voxel cluster threshold 30, *p* < 0.001, uncorrected).*

*Note that MNI coordinates are used. The *p*-values for a voxel-wise FWE-corrected threshold are shown. Stars delineate sub-clusters that are more than 8 mm from the center coordinate.*

There were no overlapping clusters, so it was not possible to calculate a percent overlap between the two groups. By simply looking at the corresponding activity maps (**Figures 4** and **5**), it is clear that the activity observed in the magician differs from the one in the experimental sample. For the magician, we found parietal and sensory-motor activity, whereas the naïve subjects had active clusters in the more anterior parts of the brain and the basal ganglia (CN). To additionally confirm this, we conducted a conjunction analysis (with the conjunction null, Nichols et al., 2005) for the contrast magic – control to identify common areas of activity between both the magician and the normal volunteers. However, no common clusters of activity between the magician and the normal volunteers were found, even at the less restrictive threshold of *p* < 0.001 with 30 consecutive voxels.

## DISCUSSION

In this study, we examined the violation of expected action-outcome sequences that are pervasive in magic tricks. When comparing magic tricks with a condition in which the action-outcome relationship was expected, we found four specific clusters of activity in the head of the CN bilaterally, the left inferior frontal gyrus and the left anterior insula. This activity was not present in the magician who had performed the tricks, and where we would not expect an expectation violation. The frontal activity was present at the moment the expected action–outcome contingency was violated, as well as throughout the entire magic clip. The CN, on the other hand, was only significantly active throughout the entire clip but not at the time point of the expectation violation.

The presence of subcortical activity may seem surprising at first, but it is now widely accepted that, in addition to their traditional role in motor processes, the basal ganglia also subserve higher cognitive functions (Middleton and Strick, 2000). The CN has been implicated in processing changes in the contingency between action and outcome for successful goal-directed action (see Grahn et al., 2008 for a review). Such changes in contingency are common in magic tricks, as illustrated in the following example from our stimulus set (Salt Vanish, see Supplementary Material): pouring salt into the closed fist of one hand and then slowly opening the fingers should let the salt trickle down on the table. The action “opening fingers” starts at once an internal simulation (e.g., Wolpert and Flanagan, 2001; Grush, 2004) that results in an expected outcome, namely the salt trickling down. This outcome expectation is violated when the salt vanished. As discussed in the Section “Materials and Methods,” the main difference between the two conditions in the present study (magic and control) is the expectation violation that is present in the magic clips but completely missing in the latter. We argue that in the present study, the head of the CN is bilaterally activated due to the expectation of an incongruency between the observed action and the presented outcome. The CN was not significantly active when only the discrete time point of expectation violation was analyzed; rather it was active throughout the entire magic clip. This suggests that the CN is involved in expectation rather than the incongruency itself. This is reasonable if we assume that in order to experience any violation in an expected action-outcome congruency, this expectation must build up during the preceding action sequence that leads to the unexpected outcome.

The present findings fit to a previous study that reported the CN to signal “breaches of expectation” (Schiffer and Schubotz, 2011). In contrast to the majority of studies (see Diekhof et al., 2012; or Sescousse et al., 2013 for recent reviews), they investigated caudate activity not in the context of conditional learning and reward, but under the assumption that the CN signals violations of expectations in general, independent of feedback. Schiffer and Schubotz (2011) used a movement observation paradigm (watching the movements of a dancer, with unexpected deviations from a previously learnt choreography), which can be compared to observing the magician’s unexpected movements.

We believe our results suggest a specific role of the CN during the observation of magic tricks in signaling the expectation of a violation in an action-outcome sequence, together with the prefrontal cortex (PFC). The PFC is thought to subserve the ability to select actions or thoughts to achieve internal goals, based upon a hierarchy of cognitive function along the anterior–posterior axis of the lateral PFC (Koechlin and Summerfield, 2007). In this model of executive function, decisions between multiple prior cues occur at the most anterior part of the PFC, whereas the posterior PFC is responsible for interpreting immediate environmental cues for action selection. A recent study showed that this hierarchy is reflected in the cortico-subcortical loop (Jeon et al., 2014). Branching and episodic control of action activated the ventrolateral PFC (BA45) in a region very similar to the area activated in our study and this region was connected to the anterior region of the head of the CN, where we also see activity. A meta-analysis of 126 PET and fMRI studies uncovered substantial functional connections between the left CN and the left inferior frontal gyrus (Postuma and Dagher, 2006). This means, across a large number of studies and tasks, both regions tended to be simultaneously active. Although there were no explicit task demands in our study, it seems plausible that observing a magic trick involves the conceptualization and expectation of possible action-outcomes, which relies on the information processing in the PFC and CN. This interplay is consistent with the activity in both of these regions throughout the entire magic clip, with an additional increase in PFC activity during the moment of expectation violation.

The inferior frontal gyrus activity that we found may to some degree reflect the processing of surprise. Since our study was designed to increase statistical power with a larger number of clips, we did not implement a condition controlling for surprise and thus cannot exclude this possibility. Notably, Parris et al. (2009) report a similar region (although more ventrally) underlying surprise processing. That we found inferior frontal gyrus activity when exclusively looking at the moment of magic points into that direction, too. But it is difficult without further experiments, or perhaps a future meta-analysis, to know whether the inferior frontal region found by Parris et al. (2009) is the same region found here and whether this corresponds to an overlapping underlying cognitive process. We are just beginning to understand the subdivisions and cognitive functions attributed with these regions.

The anterior insula has been implicated in a wide range of tasks and cognitive processes (e.g., Craig, 2009; Gasquoine, 2014). Craig (2009) pointed out that these heterogeneous findings could be subsumed under the header “awareness” and postulated that the anterior insula is a key area in human awareness and consciousness. Based on their meta-analysis of 1768 fMRI experiments, Kurth et al. (2010) suggested the anterior–dorsal insula as a multimodal integration region, because it was the only region in which nearly all of the 13 investigated functional categories (e.g., emotion, empathy, memory, interoception) overlapped. It is often found to be co-activated with the ACC, one of the regions that was also found in the Parris et al. (2009) study where ACC activity was interpreted as mirroring conflict detection mechanisms.

To a large extent, we were successful in our replication attempt of Parris et al. (2009). We also found activity in the DLPFC (superior frontal gyrus), and in parts of the cingulate cortex, when we used the same time point of the analysis. The remaining differences in activation are likely due to differences in the design, as well as in the additional condition to control for surprise that was present in Parris et al. (2009). Also, our analysis was a whole-brain analysis whereas Parris et al. (2009) analyzed specific anatomical regions of interest. One intriguing consensus between the two studies was the left-dominant activity in the PFC. The left PFC, in particular the DLPFC, is thought to be involved in interpreting complex actions (Gazzaniga, 2000; Roser et al., 2005). A previous study on causality violation also found left-dominant DLPFC activity, which they associated with reasoning and interpreting the observed events (Fugelsang and Dunbar, 2005). Our results agree with the previous findings.

As hypothesized, the magician’s brain activity differed clearly from the experimental group. It was mainly parietal activity, whereas the experimental group had active clusters in the more anterior parts of the brain and the basal ganglia. That we did not find any overlapping regions in our conjunction analysis shows that the magician processed the magic tricks and the control clips differently than lay people and supports our hypothesis that he did not experience any expectation violations. The most prominent cluster was centered in the supramarginal gyrus bilaterally. Recently, the right supramarginal gyrus was proposed to subserve self–other distinction in a paradigm investigating the emotional egocentricity bias (Silani et al., 2013). In that study, the right supramarginal gyrus was implicated in overcoming emotional egocentricity. Since the magician watched himself in the videos, but was fully aware that other people would be watching the clips, too, it seems plausible that he was trying to see himself with other people’s eyes. However, it is not clear to which extent emotions played a role in the current paradigm, neither for the experimental group nor for the magician, because this was not assessed.

Of course, a comparison between a group and a single subject, as performed in this work, is methodologically dissatisfying. However, for the question we were trying to tackle, namely how the magic tricks would be perceived by someone who knew the action sequences very well and would thus not experience any expectation violations, it is difficult to conceive of a better method. Even testing more magicians (apart from the difficulties in recruiting them) would not have improved the design, since they had not performed the same tricks. Of course, they might know many of the tricks, but still perform them in a different manner and thus not be able to represent and predict the entire action sequence as well as Thomas Fraps. Thus, it seems difficult to imagine actually testing a collective. A potential improvement would be to have, e.g., five magicians, and all of them perform five tricks. That is, in the test condition they will watch 5 self generated and 20 other generated tricks.

Clearly, the idea of expectation violation in magic tricks can be related to the concept of prediction errors. A magic effect is a non-predictable event. The anterior insula, one of the active clusters found, is thought to process prediction errors and risk (e.g., Bossaerts, 2010). Although prediction errors are typically investigated in the context of gambling tasks where participants make actual decisions, based on their predictions about possible outcomes of their decision, this could be transferred to the present situation in which participants might have predicted the outcome of the observed action – and experienced a prediction error in the case of an unexpected outcome (i.e., in the magic clips, but not in the control clips). That we also found activity in the inferior frontal gyrus, a region implicated in risk prediction error processing and closely connected with the anterior insula (Bossaerts, 2010), supports this view. Leaving the context of risk and reward processing, and focusing on a more general prediction mechanism, Zacks et al. (2007) have introduced the terms “perceptual predictions” and “perceptual prediction error” in their theory of event prediction. This might provide a useful framework to further investigate the special type of expectation violation in magic tricks that was the focus of the present work.

## Conflict of Interest Statement

The authors declare that the research was conducted in the absence of any commercial or financial relationships that could be construed as a potential conflict of interest.
