# Supplementary material for: An fMRI investigation of expectation violation in magic tricks
Source: Front Psychol. 2015 Feb 4;6:84. doi: 10.3389/fpsyg.2015.00084 (PMC4316608; doi:10.3389/fpsyg.2015.00084)
Supplement: Supplementary file 1 [file Data_Sheet_1.DOCX]

**Supplementary Material**

List of the 24 stimuli used.

| **Name** | **Magic effect** | **Description of magic clip** | **Description of control clip** | **Surprise (1-4) ^a^** | **Illusion (1-4) ^b^** | **Viola-tion of law of cause and effect (1-4) ^c^** | **Un-expected outcome (1-4) ^d^** |
| --- | --- | --- | --- | --- | --- | --- | --- |
| Orange to Apple | Transformation | Magician takes an orange from a fruit bowl. He throws it up into the air, catches it again and it has turned into an apple. | Magician takes an orange from a fruit bowl. He throws it up into the air, catches it again and it is still an orange. | 3.80 | 3.60 | 3.67 | 3.80 |
| Bottled Scarf | Vanish | Magician pushes a red silk scarf into a milk bottle, puts the lid on and shakes the bottle. The red scarf disappears. | Magician pushes a red scarf into a milk bottle, puts the lid on and shakes the bottle. The red scarf remains in the bottle. | 3.47 | 3.40 | 3.47 | 3.33 |
| Ketchup Bottle | Vanish | A ketchup bottle is presented and put into a brown bag. Magician crumples the brown bag into a small paper ball. | A ketchup bottle is presented and put into a brown bag. Magician takes the bottle out before crumpling the brown bag into a small paper ball. | 3.27 | 3.53 | 3.67 | 3.13 |
| Cigarette | Vanish | Magician tries to light a cigarette. While fumbling with the lighter, the cigarette disappears. While he looks at his empty hand, the lighter disappears, too. | Magician tries to light a cigarette. He fumbles with the lighter, looks at the cigarette, but none of them disappears. | 3.27 | 3.40 | 3.13 | 3.47 |
| Spoon | Transformation | A spoon is put into the magician's mouth and reappears as a fork. | A spoon is put into the magician's mouth and reappears as a spoon. | 3.13 | 3.27 | 3.40 | 3.00 |
| Rubik's Cube | Transformation | Magician shows a disarranged Rubik's Cube, throws it into the air and catches it, perfectly arranged. | Magician shows a disarranged Rubik's Cube, throws it into the air and catches it, still disarranged. | 3.13 | 3.67 | 3.40 | 2.87 |
| Coat Hanger | Topological impossibility (size) | Magician shows a small purse. He opens it and takes out the hook of a coat hanger. He then looks into the purse again and pulls the large wooden part of the coat hanger out. | Magician shows a small purse. He opens it and takes out a hook. He then looks into the purse again and takes the large wooden part from the table. | 3.07 | 3.00 | 3.20 | 2.87 |
| Traveling Coins | Transposition | Magician shows 3 coins in each hand. He closes his hands. For each hand separately, he drops the coins on the table, counts them and takes them back. After shaking both fists, he opens them to reveal that all 6 coins are now in one hand. | Magician shows 3 coins in each hand. He closes his hands. For each hand separately, he drops the coins on the table, counts them and takes them back. After shaking both fists, he opens them and the coins are still in their place, 3 in each hand. | 3.07 | 3.40 | 3.33 | 2.67 |
| Restora-tion | Restoration | Magician takes a card from the deck, rips it in several pieces and puts these on the deck of cards on the table. He then slams down his hand on the pack and lifts it up again to reveal the restored card. | Magician takes a card from the deck, rips it in several pieces and puts these on the deck of cards on the table. He then slams down his hand on the pack and lifts it up again to reveal the ripped pieces. | 3.07 | 3.53 | 3.60 | 2.93 |
| Floating Ball | Telekinesis (Levitation) | Magician holds a ball between his fingers. He lets go of the ball and it floats in the air, moving slowly up and down. He moves a hoop around the ball to show that no strings are attached to it. | Magician holds a ball between his fingers. He moves it slowly up and down. | 3.07 | 3.20 | 3.20 | 2.33 |
| Paper to Money | Transformation | Paper sheets are folded in half. Upon unfolding, it is revealed that they have turned into 50 Euro bills. | Paper sheets are folded in half. Upon unfolding, they are still the same. Magician takes folded 50 Euro bills from his shirt pocket and unfolds them, too. | 3.00 | 3.27 | 3.00 | 2.60 |
| Billiard Balls | Appearance | Magician shows hand empty. Then he takes a red ball in his hand, waves it in the air and a second ball appears. | Magician shows hand empty. Then he takes a red ball in his hand, waves it in the air and nothing happens. Out of his shirt pocket, he pulls a second ball. | 2.93 | 3.07 | 2.87 | 2.67 |
| Penetra-ting Coin | Penetration | A coin is put onto a glass that is sealed with a grey rubber foil. The magician presses down on the coin until it falls into the glass, but the foil remains intact. | A coin is put onto a glass that is sealed with a grey rubber foil. The magician presses down on the coin, but it doesn't penetrate the foil and doesn't fall into the glass. | 2.87 | 3.13 | 3.27 | 3.00 |
| One-Handed Coin Vanish | Appearance and Vanish | A coin is held up in the air, magician closes his fist around it. He opens the hand and the coin has disappeared. | A coin is held up in the air, magician closes his fist around it. He opens the hand and the coin is still there. | 2.80 | 3.20 | 3.07 | 3.00 |
| Colour Changing Card | Transformation | Magician puts a pack of cards into a glass. He holds the glass in the air, pulls a red silk scarf over the cards, shortly covering them, and when they are visible again, the front card has changed its colour. | Magician puts a pack of cards into a glass. He holds the glass in the air, pulls a red silk scarf over the cards, shortly covering them, and when they are visible again, the front card is still the same. | 2.80 | 2.67 | 2.73 | 2.87 |
| Knive Transposi-tion | Transposition | Magician presents two knives of different colour. He puts the red one in his left hand, and the white one in his right hand. He closes both hands and opens them to reveal that the knives have changed places. | Magician presents two knives of different colour. He puts the red one in his left hand, and the white one in his right hand. He closes both hands and opens them to reveal that the knives have remained in the same place. | 2.80 | 3.27 | 3.20 | 2.87 |
| Vanishing Coin | Vanish | Magician has 3 coins in his hand. He closes the hand, shakes it and opens it to reveal that only 2 coins are left. | Magician has 3 coins in his hand. He closes the hand, shakes it and opens it to reveal all 3 coins. | 2.80 | 3.27 | 3.20 | 2.87 |
| Bouncing Egg | Physical impossibility | Sitting at a table, the magician takes an egg from an egg box. Behind the table, he throws it to the floor a few times – and it always jumps back into his hands, undamaged. He then breaks it and empties the content into a glass. | Sitting at a table, the magician takes an egg from an egg box. Behind the table, he slowly moves it down towards the floor and up again. He then breaks it and empties the content into a glass | 2.73 | 2.80 | 3.00 | 2.67 |
| Salt Vanish | Vanish | A large amount of salt is poured into the fist. Magician blows on the fist, opens it and the salt has disappeared. | A large amount of salt is poured into the fist. Magician blows on the fist, opens it and the salt is still there. | 2.67 | 2.80 | 2.93 | 2.53 |
| Triumph | Telekinesis | A pile of upward facing cards is riffle-shuffled into a pile of downward facing cards. The complete deck of cards is spread out to show that it contains both upward and downward facing cards. The deck is then turned over, the magician snaps his fingers, and spreads all cards across the table. All cards are now facing downward. | A pile of upward facing cards is riffle-shuffled into a pile of downward facing cards. The complete deck of cards is spread out to show that it contains both upward and downward facing cards. The deck is then turned over, the magician snaps his fingers, and spreads all cards across the table - and nothing has changed. | 2.67 | 3.27 | 3.27 | 2.80 |
| Bowling Ball | Topological impossibility (size) | Magician enters the room carrying only a thin suitcase. He puts it on a small table, opens it and produces a large bowling ball. | Magician enters the room carrying the suitcase and the bowling ball together, one in each hand. He puts both on the table. | 2.60 | 3.07 | 3.20 | 2.47 |
| Salt 'n Pepper | Vanish | Salt and pepper are poured into the palm of one hand. Magician closes the hand, shakes it and opens it to reveal that the pepper has disappeared. | Salt and pepper are poured into the palm of one hand. Magician closes the hand, shakes it and opens it to reveal both pepper and salt. | 2.60 | 2.67 | 2.53 | 2.47 |
| Three Card Monte | Transposition | 3 cards (2 black ones and a red one) are presented and put on the table facing downwards. The magician turns them over and the red card has moved to the place of the black card. | 3 cards (2 black ones and a red one) are presented and put on the table facing downwards. The magician turns them over and the red card stays in its position. | 2.53 | 2.60 | 2.80 | 2.67 |
| Traveling Coin | Transposition | Magician presents a napkin and a coin. He puts both down on the table, then grabs the coin and transfers it to the other hand that closes around it. He opens the hand to reveal that the coin has vanished. He lifts the napkin to show that the coin is lying underneath the napkin. | Magician presents a napkin and a coin. He puts both down on the table, then grabs the coin and transfers it to the other hand that closes around it. He opens the hand to reveal that the coin is still there. | 2.33 | 2.60 | 2.67 | 2.67 |

In a pilot study, all clips were rated on a scale from 1 (not at all) to 4 (very much) by 15 naïve participants. Mean ratings are shown for ^a^ how surprising the clip was ^b^ how much it involved illusion  ^c^ how much it violated the law of cause and effect  ^d^ whether the magician’s actions led to an unexpected outcome. Stimuli are sorted according to the mean rating of surprise
